# Participation of Myosin Va and Pka Type I in the Regeneration of Neuromuscular Junctions

**DOI:** 10.1371/journal.pone.0040860

**Published:** 2012-07-16

**Authors:** Ira Verena Röder, Siegfried Strack, Markus Reischl, Oliver Dahley, Muzamil Majid Khan, Olivier Kassel, Manuela Zaccolo, Rüdiger Rudolf

**Affiliations:** 1 Institut für Toxikologie und Genetik, Karlsruhe Institute of Technology, Eggenstein-Leopoldshafen, Germany; 2 Institut für Angewandte Informatik, Karlsruhe Institute of Technology, Eggenstein-Leopoldshafen, Germany; 3 Institute of Neuroscience and Psychology, University of Glasgow, Glasgow, United Kingdom; 4 Institut für Medizintechnologie, Universität Heidelberg und Hochschule Mannheim, Mannheim, Germany; 5 Institut für Molekular- und Zellbiologie, Hochschule Mannheim, Mannheim, Germany; University of Rome La Sapienza, Italy

## Abstract

**Background:**

The unconventional motor protein, myosin Va, is crucial for the development of the mouse neuromuscular junction (NMJ) in the early postnatal phase. Furthermore, the cooperative action of protein kinase A (PKA) and myosin Va is essential to maintain the adult NMJ. We here assessed the involvement of myosin Va and PKA in NMJ recovery during muscle regeneration.

**Methodology/Principal Findings:**

To address a putative role of myosin Va and PKA in the process of muscle regeneration, we used two experimental models the dystrophic mdx mouse and Notexin-induced muscle degeneration/regeneration. We found that in both systems myosin Va and PKA type I accumulate beneath the NMJs in a fiber maturation-dependent manner. Morphologically intact NMJs were found to express stable nicotinic acetylcholine receptors and to accumulate myosin Va and PKA type I in the subsynaptic region. Subsynaptic cAMP signaling was strongly altered in dystrophic muscle, particularly in fibers with severely subverted NMJ morphology.

**Conclusions/Significance:**

Our data show a correlation between the subsynaptic accumulation of myosin Va and PKA type I on the one hand and NMJ regeneration status and morphology, AChR stability and specificity of subsynaptic cAMP handling on the other hand. This suggests an important role of myosin Va and PKA type I for the maturation of NMJs in regenerating muscle.

## Introduction

**Figure 1:**
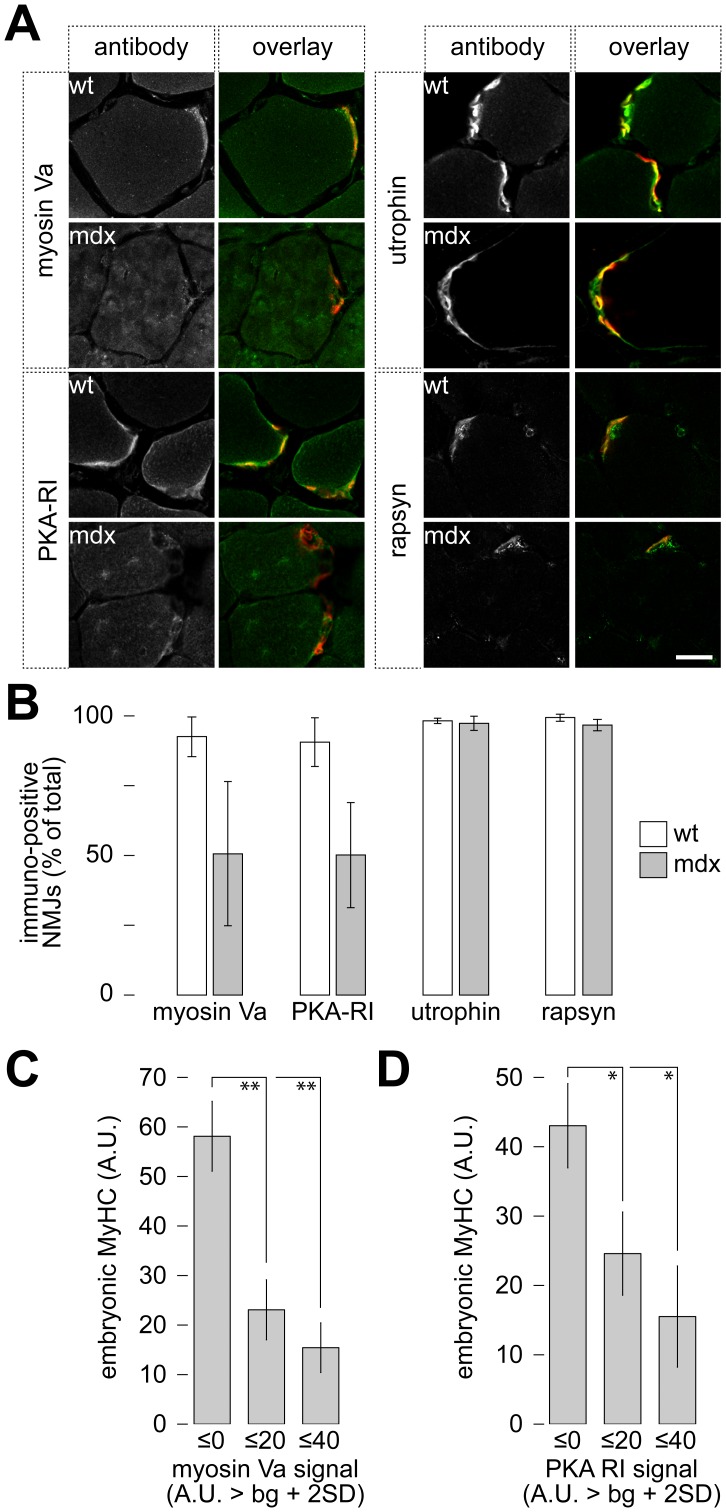
Subsynaptic accumulation of myosin Va and PKA-RI differs between wildtype and dystrophic mice and correlates with fiber diameter. Tibialis anterior muscles were snap-frozen, sliced transversally and immunostained. BGT-AF647 (A–B) or BGT-AF555 (C–D) was used to label NMJs. A: Representative confocal images showing immunofluorescence signals against either myosin Va, PKA-RI, utrophin, or rapsyn in green, and NMJs in red. In overlay images colocalizing signals appear yellow. Mouse strains as indicated. Scale bar, 20 µm. B: Quantification of the accumulation of the different proteins within the NMJ region. Data represent mean ± SEM (n ≥ 3 muscles). C–D: Muscles were co-immunostained against embryonic myosin heavy chain and either myosin Va (C) or PKA type I (D). Depicted is the amount of embryonic myosin heavy chain staining intensity as a function of subsynaptic accumulation of myosin Va (C) and PKA-RI (D). Data represent mean ± SEM (n = 6 for myosin Va and n = 4 for PKA).

The vertebrate neuromuscular junction (NMJ) is the cholinergic synapse between motor neurons and skeletal muscle fibers. On their postsynaptic side NMJs exhibit an extremely high density of about 10,000 nicotinic acetylcholine receptors (AChRs) per square micron of synaptic membrane. AChRs are normally aligned in continuous, winding band-like arrays, which often assume a “pretzel”-like shape [1], [2]. AChRs mediate neurotransmission and induce nerve-evoked voluntary muscle contraction. As typical transmembrane proteins, they pass the endoplasmic reticulum, the Golgi apparatus, and exocytic carriers to reach the plasma membrane [3], [4]. From there, receptors can be endocytosed and degraded [5], [6], presumably by entering the lysosomal compartment [7], [8], [9], [10]. However, another pool of AChRs can be recycled back to the plasma membrane in an activity-dependent manner [8], [11], [12], [13], [14], [15], [16]. Indeed, radio-labeling experiments have shown that in mice AChRs might exhibit three distinct half-lives, i.e. roughly one day, one week, or two weeks, depending on muscle activity [5], [17], [18], [19]. It is unclear, if that reflects the number of recycling passages and how such distinct lifetimes can be achieved. However, in search of possible factors mediating AChR stabilization, previous reports suggested the involvement of cAMP and protein kinase A (PKA) signaling [20], [21], [22], [23]. Our own data corroborated an involvement of PKA type I in the process of AChR stabilization [14] and showed that for that purpose rapsyn is crucial to anchor PKA type I in close proximity to the NMJ [24]. Furthermore, myosin Va, a two-headed actin-dependent motor protein [25], [26], cooperates with PKA type I for stabilizing AChRs [14] and for proper myosin Va function an intact, well-developed subsynaptic actin-cytoskeleton is instrumental [14]. Notably, muscles lacking the actin-organizing protein, dystrophin, like those from the Duchenne muscular dystrophy mouse model, mdx, exhibit severely altered NMJ morphology and reduced metabolic lifetime of AChRs [27], [28], [29]. The latter was rescued by means of cAMP agonists, further supporting a role of cAMP/PKA-dependent signaling in AChR lifetime regulation [28]. Building on these findings here we investigate a possible role of myosin Va and PKA type I in two models of muscle regeneration. Our data suggest that the degree of subsynaptic accumulations of myosin Va and PKA type I correlate with NMJ maturation and that this is important for proper AChR turnover and subsynaptic signaling.

## Results

### Myosin Va and PKA-RI are Less Abundant in Mdx NMJs

Muscles of mdx mice undergo repetitive degeneration-regeneration cycles leading to a mixed composition in muscles concomitantly displaying newly formed myotubes, immature and mature muscle fibers [30]. In case of fiber maturation-dependent enrichment of myosin Va and PKA-RI one would expect a high heterogeneity of these proteins’ subsynaptic accumulation. To test this hypothesis, tibialis anterior muscles of adult wildtype and mdx mice were cross-sectioned and then co-stained with the AChR marker, α-bungarotoxin (BGT) fluorescently labeled with AlexaFluor 647 (BGT-AF647), to identify NMJs, and with antibodies against myosin Va, PKA-RI, utrophin, or rapsyn. Then, confocal images were taken of fluorescence signals of both BGT-AF647 and immunostaining (Fig. 1A), and the amounts of NMJs exhibiting a specific enrichment for each of these proteins were determined as described in the Methods section (Fig. 1B). This analysis showed a strong reduction of myosin Va and PKA-RI in NMJs from mdx as compared to wildtype mice. Indeed, while in wildtype muscles approximately 90% of all NMJs showed a clear fluorescence signal above fiber signal, only about 50% of NMJs showed such enrichment in muscles from mdx mice. Notably, in the latter a very high variability in signal intensities was observed between different muscles, preventing statistical significance. Conversely, utrophin and rapsyn signal intensities were similar in wildtype and mutant animals. Here, close to 100% of NMJs were immunopositive in all investigated muscles. To test whether myosin Va and PKA-RI are particularly enriched in the subsynaptic regions of mature fibers, we capitalized on three characteristics of mdx muscles, i.e. re-expression of embryonic myosin heavy chain in regenerating fibers [31], occurrence of center-nucleated fibers that have undergone regeneration [32], and increased fiber diameter variability [32]. First, we correlated the abundance of the regeneration marker, embryonic myosin heavy chain [33], [34], which was present in newborn and adult mdx muscles, but absent from adult wildtype muscles (Fig. S1), with the subsynaptic enrichment of myosin Va and PKA-RI. This revealed that fibers with high expression levels of embryonic myosin heavy chain displayed low enrichment of myosin Va and PKA-RI at the NMJ, and vice versa (Fig. 1C and D). Second, we correlated the subsynaptic enrichment of myosin Va and PKA type I with the occurrence of central positioning of fibers. In mdx, central location of nuclei is a persistent feature of fibers that have undergone a degeneration/regeneration cycle. Thus, it is not a direct indicator of a recent regeneration event [31]. However, fibers with central nuclei exhibited less NMJs with enrichment of myosin Va (Fig. S2A) and PKA type I (Fig. S2B) as compared to fibers with peripheral nuclei. Finally, we determined subsynaptic accumulation of myosin Va and PKA-RI together with the diameters of the corresponding fibers. This revealed a positive correlation between fiber diameter and the subsynaptic enrichment of myosin Va (Fig. S2C) and PKA-RI (Fig. S2D). Together, these data indicate that myosin Va and PKA-RI exhibit particularly low abundance in synapses of immature or regenerating fibers.

**Figure 2:**
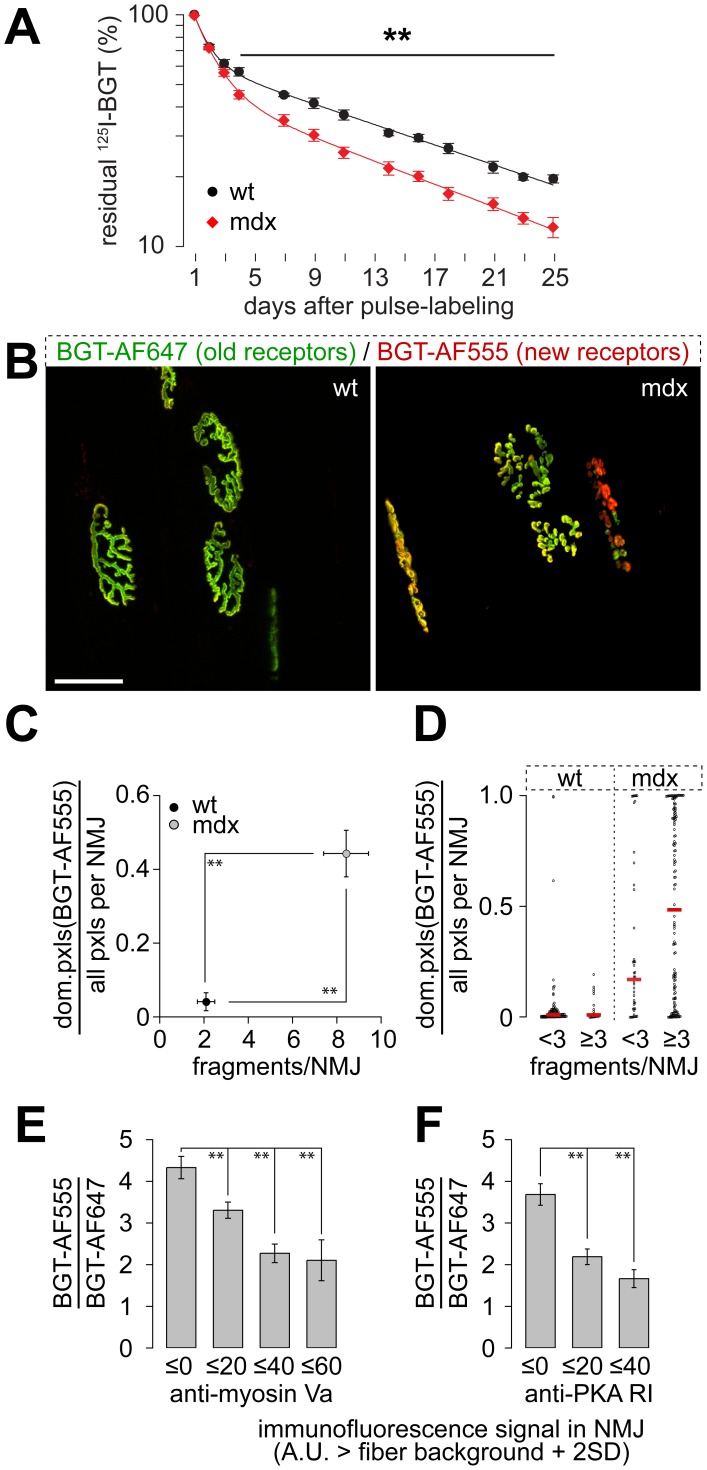
Lifetime of AChRs is reduced in dystrophic muscles and correlates with synaptic integrity and subsynaptic enrichment of myosin Va and PKA-RI. A: Tibialis anterior muscles of wildtype (wt) and mdx mice were pulse-labeled with ^125^I-BGT on day 0. Subsequently, residual ^125^I-emission was measured repetitively in the live animals at indicated time points from these muscles in situ. For the duration of measurements (10 min), mice were anaesthetized with isofluorane. Data represent mean ± SEM (n = 5 mice). Welch-test revealed significant differences between wildtype and mdx values, ** p<0.01. B–F: Tibialis anterior muscles were injected with BGT-AF647 (old receptors). Ten days later, muscles were exposed, injected with BGT-AF555 (new receptors), and then monitored with in vivo confocal microscopy (B–D). Subsequently, muscles were sliced, immunostained, and analyzed with confocal microscopy (E–F). B: Representative maximum z-projections of wildtype and mdx NMJs as indicated. Old and new receptor signals are shown in green and red, respectively. Pixels with similar intensities of both dyes appear in yellow. Scale bar, 50 µm. C: Graph showing the fraction of pixels with new receptor signals dominating over old receptor signals in individual NMJs as a function of the number of fragments per NMJ. Data represent mean ± SEM (n = 4 wildtype muscles, n = 8 mdx muscles. 109 and 191 NMJs were analyzed for wildtype and mdx, respectively). Significance was tested with Welch test, ** p<0.01. D: Graph depicts all individual values of the fractions of pixels with new receptor signals dominating over old receptor signals in NMJs. Values were grouped in NMJs with less than 3 fragments and NMJs with 3 or more fragments. Red lines indicate medians. Note the large variance in mdx. Same data set as in C. E–F: Correlations of subsynaptic myosin Va (E) and PKA-RI enrichment (F) with the apparent turnover of AChRs. Tibialis anterior muscles used for in vivo imaging (B–D) were sliced transversally and immunostained for myosin Va or PKA-RI. Ratio of old and new receptors and the accumulation of myosin Va or PKA-RI were determined for each synapse (n = 5 muscles; 651 and 340 NMJs were quantified for myosin Va and PKA-RI, respectively). Significance was tested with Welch test, ** p<0.01.

### AChR Lifetime is Diminished in Mdx Muscles

We have recently demonstrated an important role of myosin Va and PKA-RI for the stabilization of AChRs in NMJs [13], [14], [15]. Given the observed low abundance and high heterogeneity of appearance of these proteins in the NMJ regions of mdx muscles one would also expect a reduced AChR lifetime as a consequence. We addressed this question employing a recently established in vivo assay [19]. In brief, tibialis anterior muscles of adult wildtype and mdx mice were labeled with radioactive ^125^I-BGT. Subsequently, ^125^I-activity emitted from these hindlegs was measured over the next four weeks. This showed a clearly reduced AChR lifetime in mdx compared to wildtype mice (Fig. 2A).

Since the radio iodine-based assay measures the entire NMJ populations of the observed muscles, it does not allow to draw conclusions on individual synapses. We therefore used an imaging-based assay [13], [14], [19] to determine AChR turnover in individual synapses. Tibialis anterior muscles were injected with infrared fluorescent BGT-AF647 (Fig. 2B, shown in green) and ten days later with red fluorescent BGT-AF555 (Fig. 2B, shown in red). After the second injection, muscles of the anaesthetized animals were monitored with in vivo microscopy (Fig. 2B). This clearly confirmed the radio iodine-based data showing an increased turnover of AChRs in mdx as compared to wildtype muscles (Fig. 2B–C). Furthermore, in mdx mice AChR stability was mainly varying between different synapses, but not so much within a given synapse (Fig. 2B). We also observed that NMJs were more fragmented in dystrophic than in wildtype muscles (Fig. 2B–C). To study whether AChR turnover in individual synapses correlated with their morphological integrity, we separated all identified NMJs into two groups: i) synapses with intact structure (one or two continuous AChR segments) and ii) fragmented synapses with three or more AChR segments. In wildtype muscles both groups of NMJs exhibited a very low AChR turnover (Fig. 2D, median of the ratio of dominant BGT-AF555 pixels/all pixels per NMJ was 0.01 for both intact and fragmented NMJs). In mdx mice the situation was more complex: while intact synapses exhibited on average a relatively low AChR turnover (median 0.17), AChRs in fragmented NMJs were apparently much less stable (median 0.48). This strongly suggests that the increased AChR turnover participates in the fragmentation of NMJs observed in mdx muscle.

### AChR Stabiliy in Mdx NMJs is Positively Correlated with the Presence of Myosin Va and PKA-RI

Next, we asked whether the heterogeneity in AChR stability in mdx muscles might be due to the observed variability in subsynaptic enrichment of myosin Va and PKA-RI. To address this point old and newly formed AChRs were labeled with BGT-AF647 and BGT-AF555, respectively, as described before. Then, these muscles were resected and cross-sections immunostained against myosin Va or PKA-RI. Subsequently, AF647, AF555 and immunostaining fluorescence signals were measured with confocal microscopy. A quantitative analysis revealed positive correlations between AChR stability and the intensities of the subsynaptic immunofluorescence signals of myosin Va and PKA-RI (Fig. 2E–F). This suggests that AChR stability is linked to the local, subsynaptic enrichment of these two proteins in dystrophic mdx mice.

### Fragmented Mdx NMJs Show Aberrant CGRP-dependent cAMP Signals

**Figure 3:**
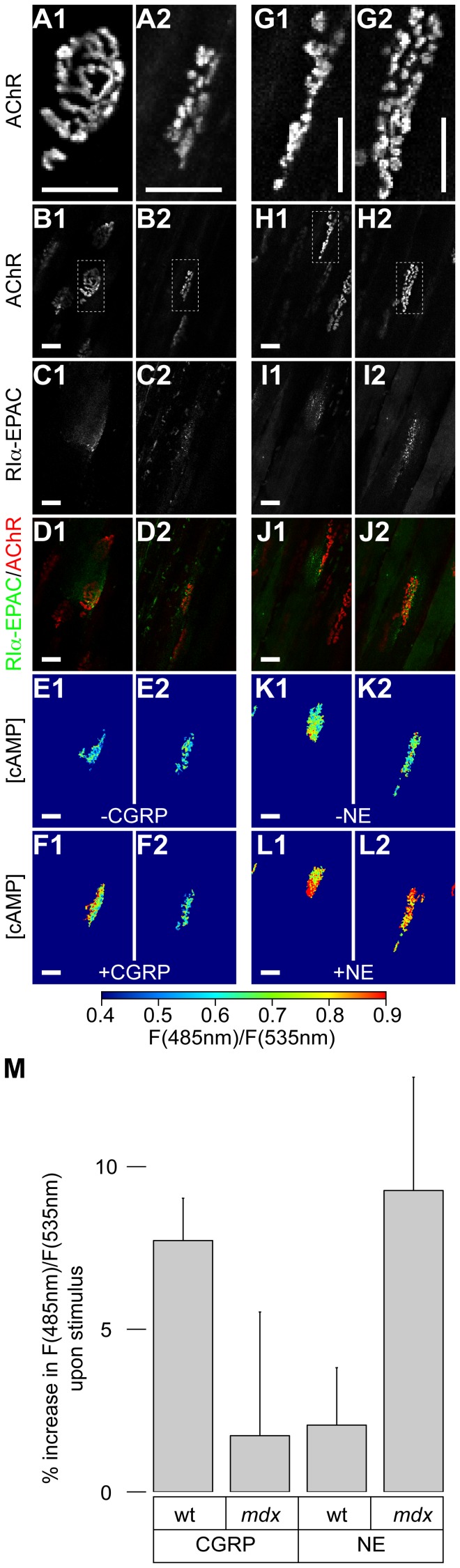
Response to cAMP agonists differs between wildtype and mdx synapses. Tibialis anterior muscles of wildtype and mdx mice were transfected with RIα-EPAC. Ten days later, muscles were injected with BGT-AF647 to stain NMJs and monitored with in vivo confocal (A–D, G–J) or two-photon (E–F, K–M) microscopy. Scale bars depict 50 µm. **A and G:** Shown are single mdx NMJs with normal (A1) or fragmented morphology (A2, G1, G2). **B and H:** BGT-AF647 fluorescence signals. Boxed regions are shown enlarged in A and G. **C and I:** RIα-EPAC fluorescence signals in the same field as in B and H. **D and J:** Overlays of B and C (D) and H and I (J). BGT-AF647 and RIα-EPAC signals are in red and green, respectively. **E and K:** Same field as in B and H showing FRET-ratios in pseudo-colors before application of CGRP or NE (indicated). **F and L:** Same field as in E and K showing FRET-ratio in pseudo-colors after application of CGRP or NE (indicated). **M:** Quantification of several experiments. Shown is the percentage of increase in CFP/YFP ratio values (F(485 nm)/F(535 nm)) compared to basal upon application of 50 µl of either 10 µM CGRP or 10 µM NE as indicated. Data represent mean ± SEM (n = 10 and n = 14 wildtype NMJs for CGRP and NE, respectively; n = 13 and n = 9 mdx NMJs for CGRP and NE, respectively).

Since myosin Va and PKA-RI play an important role in the subsynaptic cAMP signaling [14], the present finding of a highly variable presence of those proteins beneath NMJs in mdx mice prompted us to investigate the organization of cAMP handling in mdx muscle. Therefore, wildtype and mdx muscles were transfected with RIα-EPAC [35], a genetically encoded cAMP FRET-sensor directed to the PKA-RIα-microdomain. Ten days later, we injected these muscles with BGT-AF647 to label NMJs and first observed them using 3D-confocal microscopy to identify the position of the synapses and determine their morphology. 3D-two-photon microscopy served, then, to visualize the basal CFP- and YFP-signals emitted by the RIα-EPAC-sensor in the same fibers. Subsequently, cAMP agonists that target either the PKA-RI or the PKA-RII microdomains in the sarcomeric region of wildtype muscles were injected. These agonists were either α-calcitonin gene-related peptide (CGRP) or norepinephrine (NE), respectively [36]. Then, muscles were left at rest for a minute, followed by monitoring the increase in the CFP/YFP-ratio indicative for the production of cAMP in the PKA type I-microdomain. This procedure allowed us to correlate cAMP signals to NMJ morphology. Figure 3A–L depicts exemplary measurements of that kind. As can be seen in panels E–F, the treatment with CGRP elicited a cAMP response only in the PKA-RIα-microdomain underlying the intact (see Fig. 3E1 and F1), but not the fragmented NMJs of mdx muscles (see Fig. 3E2 and F2). Furthermore, in contrast to wildtype muscles, NE also elicited cAMP signals in the PKA type I-microdomain (see Fig. 3K–L). This picture was verified by a quantitative analysis (Fig. 3M): compared to wildtype muscles the average cAMP-response to CGRP in the PKA-RIα-microdomain underlying NMJs was very low, albeit with high statistical variability. Conversely, most mdx NMJs responded well to NE treatment, in stark contrast to what was observed before in wildtype animals [14]. Together, these data indicate that the specificity of cAMP signaling is largely disturbed in mdx synapses. It furthermore indicates that the responsiveness of the PKA-RIα-microdomain to CGRP is heterogeneous and, at least partially, dependent on the morphological integrity of the individual synapses.

### Subsynaptic Enrichments of Myosin Va and PKA-RI Increase during Muscle Regeneration

**Figure 4:**
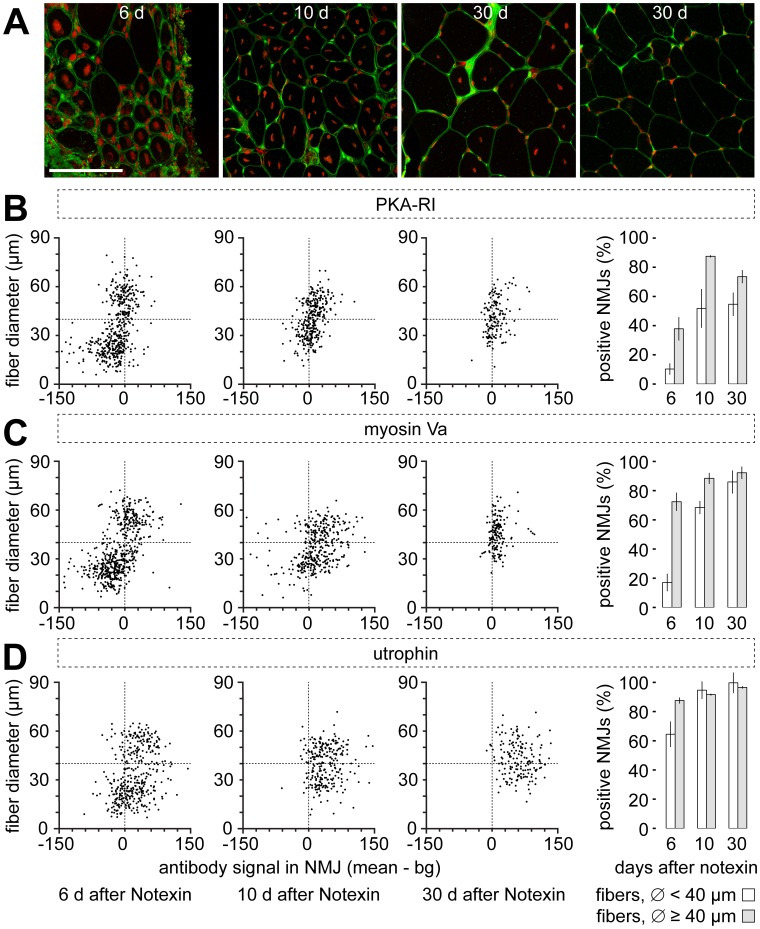
Notexin treatment transiently reduces the subsynaptic accumulation of PKA-RI and myosin Va. EDL muscles were injected with Notexin to induce muscle degeneration. 6, 10, and 30 days after treatment muscles were resected and sliced transversally. **A:** Slices were stained with wheat germ agglutinin-AlexaFluor488 for plasma membranes (green), and with DRAQ5 for nuclei (red). Images show confocal sections through muscles harvested 6, 10, or 30 days after Notexin, as indicated. Scale bar, 100 µm. **B–D:** Slices were stained with BGT-AF647 (NMJs) and with antibodies against PKA-RI (B), myosin Va (C), and utrophin (D). Confocal images were taken and then analyzed as described in the Methods section. Scatter plots (3 left columns) show diameters of all analyzed fibers as a function of their subsynaptic enrichment of immunofluorescence. Vertical dotted lines indicate the separation between immuno-negative (left halves of plots) and immuno-positive NMJs (right halves of plots). Horizontal dotted lines indicate the separation between fibers smaller and larger than 40 µm in diameter. Column graphs (right) summarize data in scatter plots and depict the fractions of immuno-positive NMJs obtained 6, 10, and 30 days after Notexin treatment. Data are mean ± SEM (n = 6, 4, and 4 muscles for 6, 10, and 30 day time points). White and grey columns represent values for fibers smaller and larger than 40 µm in diameter, respectively.

Given that in mdx mice the enrichment of myosin Va and PKA-RI at NMJs seems to correlate with the integrity of NMJs at mature fibers, one logical hypothesis is that during regeneration, these two proteins are progressively recruited to re-established synapses in maturing myofibers. To test this hypothesis, we induced muscle degeneration by Notexin injection into the extensor digitorum longus muscles of wildtype animals [37], [38]. Six, ten, and thirty days after Notexin treatment muscles were harvested and NMJs were marked with BGT-AF647 on cross-sections. Myosin Va, PKA-RI, and utrophin were immunostained. Subsequently, slices were analyzed with confocal microscopy and the fiber diameters as well as the intensities of immunostaining signals in the NMJs versus immunolabeling in the corresponding fibers were determined. Immunostaining signals were then plotted against fiber diameters. The result is depicted in Fig. 4. Six days after Notexin application there existed two populations of fibers, one with diameters of approximately 10–30 µm and a second population with diameters of 50–70 µm. The smaller-sized fibers made up about 62% of all fibers and were newly regenerating myotubes and fibers as indicated by their central positioning of nuclei (Fig. 4A). Instead, the bigger fibers were similar in size as normal ones and showed their nuclei largely in the periphery (Fig. 4A), suggesting that they were unaffected by Notexin. Already four days later, fiber size distribution was more homogeneous (Fig. 4B–D) and by day 30 after Notexin treatment there was a uniform distribution of fiber sizes ranging between approximately 30 µm and 70 µm (Fig. 4B–D). By then, most fibers exhibited again peripheral nuclei although some still showed central nuclei (Fig. 4A). While this pattern was very similar for all analyzed muscle samples the enrichment of the different marker proteins in the NMJ was not. In particular, PKA-RI and myosin Va were initially hardly present in the NMJs of smaller fibers (Figs. 4B–C). However, in the subsequent days they caught up and by day 30 they reached levels of about 60% and 90% of positive NMJs for PKA-RI and myosin Va, respectively (Fig. 4B–C). Conversely, utrophin was present in high amounts from the first phases of NMJ formation although also this protein showed a certain increment of subsynaptic enrichment during regeneration (Fig. 4D). That indicates that myosin Va and PKA-RI are recruited to regenerating NMJs in a maturation-dependent fashion. To further consolidate this aspect, we repeated the correlation analysis between expression of embryonic myosin heavy chain and subsynaptic enrichment of myosin Va in regenerating muscle ten days after injection of Notexin. Similar to the findings in mdx muscle, also here high abundance of the regeneration marker correlated with low enrichment of myosin Va and vice versa (Fig. S3).

## Discussion

We recently unraveled a crucial function of myosin Va and PKA-RI in stabilizing AChRs at the mouse NMJ [13], [14]. Furthermore, subsynaptic enrichment of myosin Va increases in parallel to the initial AChR stabilization that occurs in the early postnatal period and mice lacking myosin Va fail in stabilizing AChRs [15]. Together, these findings suggest a general association between the cooperative function of myosin Va and PKA-RI and AChR stabilization that seems to start during the first postnatal days and to persist throughout life. Here, we found that subsynaptic enrichment of myosin Va and PKA-RI correlates with the regeneration status of muscle fibers, suggesting that these proteins play a role in NMJ re-establishment during regeneration.

As a first paradigm, we studied the dystrophic mdx mouse [30], which is a well-known and yet heavily discussed model for Duchenne muscular dystrophy. These animals lack dystrophin and they exhibit many signs of muscular dystrophy, such as repetitive cycles of degeneration and regeneration of muscle fibers [30]. Although muscle wasting is less pronounced than in Duchenne patients [39] and life expectancy is almost normal, the clear genetic background and the presence of the full set of fiber maturation types, ranging from myoblasts to normal adult fibers, was ideal for this study. Indeed, we observed a high variability of the presence of myosin Va and PKA-RI in the subsynaptic region of mdx muscles, with an average of about 50% of fibers exhibiting clear subsynaptic enrichments of both markers, which inversely correlated with the expression of embryonic myosin heavy chain (Fig. 1). This was in contrast to wildtype muscles, which displayed uniformly high levels of these proteins in the range of about 90% positive fibers. Conversely, utrophin [40] and rapsyn [41] were highly enriched at all synapses of both wildtype and mdx animals. This suggests that the subsynaptic accumulation of myosin Va and PKA-RI may be partially dependent on the fiber and NMJ maturation status and may follow later during NMJ formation than utrophin and rapsyn. Notably, the high amount of synapses showing low levels of myosin Va and PKA type I, as well as fragmentation (see discussion below and Fig. 2B–D) is somehow in contrast with the finding of low regeneration levels in mdx muscles as observed in previous reports [31], [32]. This suggests that in the mdx muscles also other processes, not regeneration-dependent, may influence NMJ integrity and marker distribution. As for the enrichment of the actin-dependent motor protein, myosin Va, the absence of dystrophin itself as an actin-organizing molecule might be of relevance here. Furthermore, secondary loss of factors interacting with dystrophin could also be important in NMJ organization [42], [43], [44], [45]. Alternatively, a previous study suggested that repetitive fiber degeneration/regeneration, as well as fiber splitting and fusion in mdx muscle could be key to NMJ fragmentation [29]. However, our hypothesis of a regeneration-dependent enrichment of myosin Va and PKA type I was corroborated by the data from the Notexin-induced degeneration-regeneration experiment (Fig. 4, Fig. S3). Also here, subsynaptic accumulations of myosin Va and PKA-RI were heavily reduced few days after degeneration and recovered during regeneration, and high levels of embryonic myosin heavy chain correlated with low subsynaptic abundance of myosin Va. It should be mentioned that six days after Notexin injection we detected about 40% of fibers with normal diameter and peripheral nuclei (Fig. 4A). It is unlikely that complete fiber regeneration had taken place at that time point, because in mouse extensor digitorum longus muscle fiber degeneration is maximal three days after Notexin treatment and muscles need then several weeks for full recovery [38]. Thus, in our case, Notexin apparently did not affect the entire muscles, contrary to previous reports [38]. That might be due to experimental reasons, such as uneven distribution of the toxin in the muscle, or due to a differential sensitivity of different muscle fiber types to the toxin [37]. Thus, although the degeneration-regeneration profile was not as complete as expected, a general trend of a gradual regeneration time-dependent increase of subsynaptic accumulation of myosin Va and PKA-RI was apparent, suggesting that they indicate the NMJ maturation status.

This was further supported by the AChR lifetime analysis in mdx muscles (Fig. 2), which revealed a reduced AChR stability in dystrophic muscles and showed a direct correlation between the apparent AChR stability and the accumulation of myosin Va and PKA-RI beneath the NMJ. In other words, the more of these two proteins were found close to the synapse the more AChRs seemed to be stable. AChR stability also augmented with increasing NMJ integrity (as determined by the amount of NMJ fragments, Fig. 2D), indicating a possible link between the subsynaptic presence of myosin Va and PKA-RI on the one hand and gross synaptic integrity on the other hand.

Fragmentation of NMJs also correlates with altered cAMP signaling at the synapse. We observed three defects in subsynaptic cAMP handling of mdx muscles. First, a green fluorescent protein (GFP)-based marker of the PKA-RI microdomain, RIα-EPAC, was much less present in the subsynaptic regions of mdx muscle fibers as compared to wildtype muscles. Indeed, while in wildtype muscles 68.3% ±3.7% (n = 12 muscles) of all transfected fibers showed a clear enrichment of the marker in the NMJ area, this was true for only 23.7% ±10.8% (n = 7 muscles) of fibers in mdx. This corroborates the immunohistochemical data shown in Fig. 1. Second, when using RIα-EPAC as a FRET-based cAMP sensor as described previously [14], [35], [36], it reported in wildtype NMJs an increase in cAMP levels only upon treatment with CGRP but not with norepinephrine [14]; conversely, the majority of NMJs in mdx mice responded to norepinephrine but not to CGRP (Fig. 3M). Third, a minority of mdx NMJs also showed a rise in cAMP levels upon CGRP but that seemed to depend on their morphological integrity: NMJs with normal appearance were more likely to react to CGRP than highly fragmented NMJs. It is currently unclear why the NMJs behave so differently. A possible reason could be an altered receptor localization or activity. Alternatively, PKA-RI might not be properly tethered to its normal position, thus, getting access to the PKA-RII microdomain. This option is likely, since previous reports showed similar phenomena in the sarcomeric region of mdx fibers. In the first study, a strongly reduced PKA activity was observed although total expression levels of all PKA isoforms were not different between wildtype and mdx muscles [46]. These authors proposed, that the lack of the A-kinase anchoring protein, myospryn, in mdx muscles could be causative for the reduced PKA activity. The second work corroborated these findings by detecting a serious, A-kinase anchoring protein-dependent loss of signaling specificity in the sarcomeric region of mdx muscles [36]. Recent pharmacological data indicate that the rectification of cAMP signaling in mdx muscles using urocortins is beneficial for muscle structure and function [47]. These authors demonstrated that urocortins act via PKA and the Exchange protein directly activated by cAMP (Epac) and affect calcium homeostasis. It remains to be seen whether urocortins also ameliorate the structural integrity of NMJs in mdx muscles.

In conclusion, these new data show that subsynaptic enrichment of myosin Va and PKA-RI correlates with NMJ stabilization and/or re-establishment in regenerating muscle. This adds to and further corroborates a crucial role of the same proteins in postnatal NMJ maturation and NMJ maintenance in the adult. Our data are compatible with a model whereby myosin Va serves to bring PKA type I into close proximity of a special subsynaptic microdomain. This, in turn, might help in stabilizing AChRs and, as a consequence, gross morphology of NMJs.

## Materials and Methods

### Ethics Statement

Use and care of animals was as approved by German authorities (Tierschutzkommission of the Regierungspräsidium Karlsruhe, licenses G-88/05, G-22/07, and G-181/09) according to national law (TierSchG §§7).

### Expression Plasmids, Chemicals and Antibodies

Experiments used the cAMP sensor RIα-EPAC [35]. NE and CGRP were from Sigma. BGT-AF647 and BGT-AF555 were from Invitrogen and ^125^I-BGT was from Perkin Elmer. The nuclear marker DRAQ5, was from Biostatus Limited. Primary polyclonal antibodies: anti-PKA RIα (Cell Signaling), anti-myosin Va (LF-18, Sigma), anti-rapsyn (Mobitec) and anti-utrophin (Santa Cruz Biotechnology). Primary monoclonal antibody: anti-neonatal myosin BF-G6 (Developmental Studies Hybridoma Bank). Secondary antibodies: goat anti-rabbit-AF488 and donkey anti-mouse-AF647 (both Invitrogen). Notexin was from Latoxan (L8104).

### Animals, Transfection and Surgical Procedures

C57BL/6J, C57BL/10J and 10J Dmd^mdx^ mice were used. All animals were aged 5–7 months, except those used for Fig. 1A–B (9 weeks). Animals were from Charles River and then maintained in the local animal facility. Anaesthesia [14], and transfection [48] were as described previously. For radioactive labeling of AChRs 10 µl of an aqueous saline solution of radioactive ^125^I-BGT, containing 0.46 MBq (2,5 µCi) ^125^iodine were injected into tibialis anterior muscles of the anaesthetized mouse. The applied dose of ^125^I-BGT was about 0.1 µg or 0.01 nmol. For in vivo imaging of NMJs 25 pmol of BGT-AF647 were injected into the tibialis anterior muscles of anaesthetized mice. For muscle degeneration experiments, mice were anaethetized and then 50 µg of Notexin were injected in extensor digitorum longus muscles.

### Microscopy

All images were taken with a DMRE TCS SP2 confocal microscope equipped with Leica Confocal Software 2.61, a KrAr laser (488 nm, 514 nm), a diode-pumped laser (561 nm), a HeNe laser (633 nm), a mode-locked pulsed Maitai laser (Spectraphysics), a 63x/1.4NA PL APO OIL objective for fixed samples, a 20x/0.7NA HC PL APO CS IMM/CORR UV and a 63x/1.2NA HCX PL APO CS W CORR objective (immersion medium, Visc-Ophtal gel, Winzer-Pharma) (all Leica Microsystems) for in vivo observation. In vivo confocal microscopy, two-photon microscopy, injection of agonists and confocal microscopy of slices were performed as described previously [14]. Cryopreservation, sectioning of muscles and staining of muscle slices were done as described [14].

### Data Analysis

Image analysis employed ImageJ program (http://rsb.info.nih.gov/ij/). Accumulation of proteins within the NMJ region as shown in Figs. 1B, 2E–F, and 4 was quantified as follows: Images were median filtered (1 pixel kernel), background subtracted and thresholded from 30–255 greyscale value. The NMJ region was selected in the BGT-AF647 channel and saved as region of interest. In the immunostaining (AF488) channel the fiber area corresponding to a given NMJ was selected, and for both (NMJ and fiber) regions of interest mean grey values and standard deviations were determined. The NMJ was counted as positive with respect to an accumulation of stained protein when the mean grey value within the NMJ region was higher than the mean grey value within the fiber plus two times the standard deviation within the fiber. In formula: {(mean grey value NMJ) − (mean grey value fiber +2 standard deviations fiber)}. Correlation analysis between expression of neonatal myosin and the subsynaptic enrichment of myosin Va and PKA was done accordingly. Here, NMJs were stained with BGT-AF555, PKA or myosin Va were marked with AF488-coupled and neonatal myosin with AF647-coupled secondary antibodies. In brief, after determination of the NMJ region of interest in the AF555 channel, the corresponding fiber region was outlined in the AF647 channel. Finally, from all regions of interest mean grey values and standard deviations were determined for AF488 and AF647 channels. Analysis of RIα-EPAC localization in vivo, analysis of ‘old receptor’ and ‘new receptor’ signal densities and fragmentation and data-analysis of ratiometric videos were performed as described [14]. Analysis of AChR half-life using ^125^I-BGT was performed as described [19].

### Statistics and Graphics

Numeric data were handled using Microsoft Excel 2004 for Mac, v. 11.5, or SigmaPlot (Systat software Inc.). All data shown in graphs represent mean ± SEM unless otherwise stated. Significance was tested using Welch test. Data sets were tested for Normality using Kolmogorov-Smirnov-Lilliefors test and for homo/heteroscedasticity using F-test. Significance levels are indicated in figures or in the text (p≤0.05,*; p≤0.01,**). For data compilation, Adobe Photoshop CS2, version 9 and Adobe Illustrator CS2, version 12.0.0 were employed.

## Supporting Information

Figure S1 **Embryonic myosin heavy chain is expressed in muscles from neonates and adult mdx mice but not from adult wildtypes.** EDL muscles from wildtype newborn (A), wildtype adult (B) or from mdx adult mice (C) were snap-frozen and then stained using BGT-AF555 to label AChRs (green signals) and antibody BF-G6 against embryonic myosin heavy chain (red signals). Panels show single optical sections. Scale bar, 50 µm.
(TIF)

Figure S2 **Subsynaptic enrichments of myosin Va and PKA type I correlate inversely with the occurrence of central nuclei and positively with fiber diameter.** TA muscles from adult mdx mice were immunostained against myosin Va (A and C) or PKA type I (B and D). Synapses were labeled with BGT-AF555. A–B: Muscles were also stained with the nuclear marker, DRAQ5, imaged with confocal microscopy and then quantitatively analyzed using Image J. The graph shows the percentage of fibers with central nuclei as a function of the subsynaptic enrichment of immunostaining. NMJ regions with immunostaining > sarcomeric immunostaining signal + 2*SD were counted as positive (pos), all others as negative (neg). Data represent mean ± SEM (n = 4 muscles). C–D: Fiber diameter as a function of subsynaptic accumulation of myosin Va and PKA-RI. Data represent mean ± SEM (n = 4 muscles).
(TIF)

Figure S3 **Embryonic myosin heavy chain expression inversely correlates with subsynaptic enrichment of myosin Va in regenerating EDL muscles.** EDL muscles from adult wildtype mice were injected with Notexin. Ten days later, muscles were harvested, snap-frozen and then co-stained against embryonic myosin heavy chain and myosin Va. Synapses were labeled with BGT-AF555. Muscles were imaged with confocal microscopy and then quantitatively analyzed usinFg Image J. Depicted is the amount of embryonic myosin heavy chain staining intensity as a function of subsynaptic accumulation of myosin Va. Data represent mean ± SEM (n = 5).
(TIF)
