# Supplementary figures and images for: Participation of Myosin Va and Pka Type I in the Regeneration of Neuromuscular Junctions
Source: PLoS One. 2012 Jul 16;7(7):e40860. doi: 10.1371/journal.pone.0040860 (PMC3397957; doi:10.1371/journal.pone.0040860)

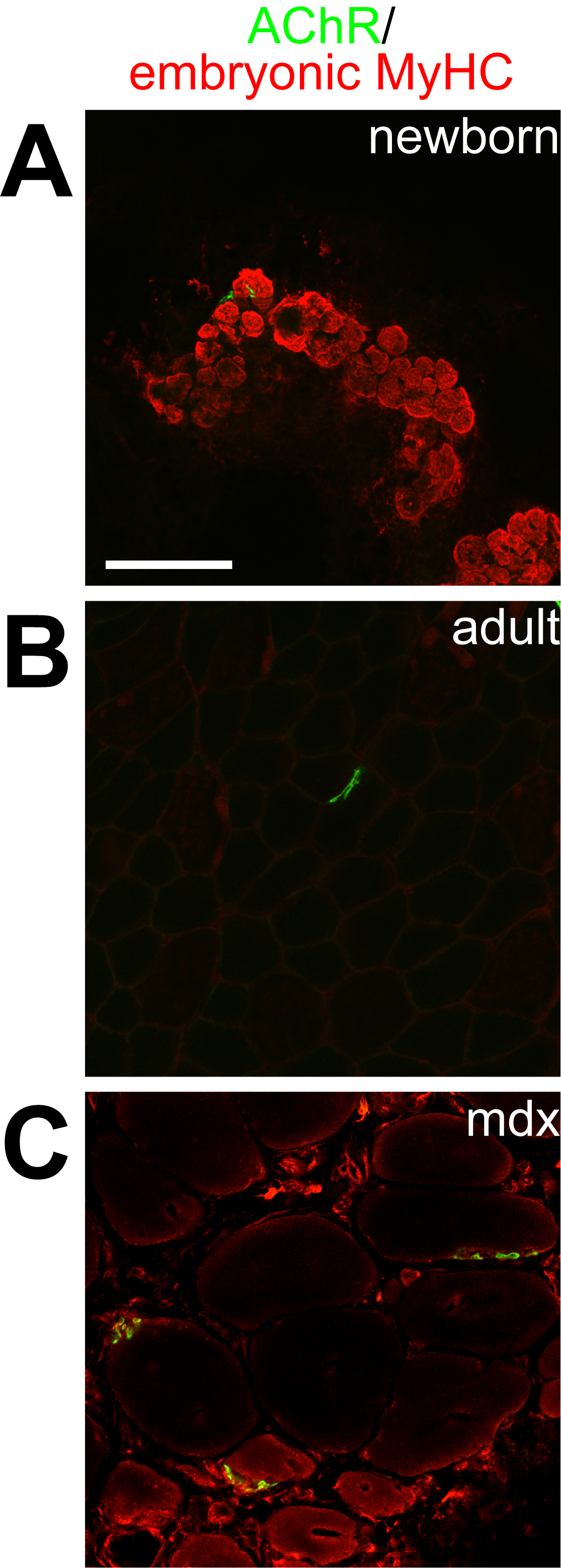

Supplement: Figure S1 — Embryonic myosin heavy chain is expressed in muscles from neonates and adult mdx mice but not from adult wildtypes. EDL muscles from wildtype newborn (A), wildtype adult (B) or from mdx adult mice (C) were snap-frozen and then stained using BGT-AF555 to label AChRs (green signals) and antibody BF-G6 against embryonic myosin heavy chain (red signals). Panels show single optical sections. Scale bar, 50 µm. (TIF) [file pone.0040860.s001.tif]

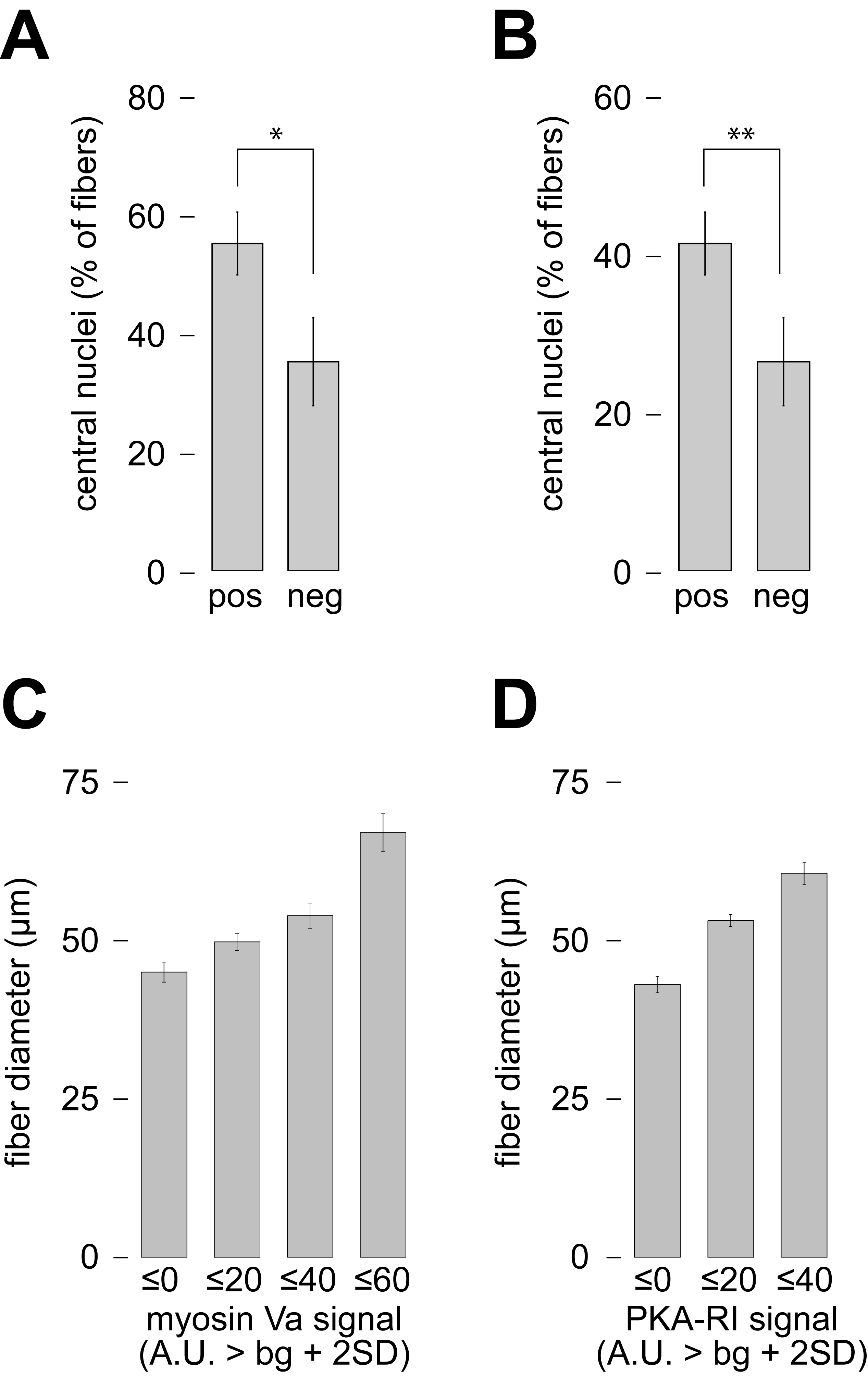

Supplement: Figure S2 — Subsynaptic enrichments of myosin Va and PKA type I correlate inversely with the occurrence of central nuclei and positively with fiber diameter. TA muscles from adult mdx mice were immunostained against myosin Va (A and C) or PKA type I (B and D). Synapses were labeled with BGT-AF555. A–B: Muscles were also stained with the nuclear marker, DRAQ5, imaged with confocal microscopy and then quantitatively analyzed using Image J. The graph shows the percentage of fibers with central nuclei as a function of the subsynaptic enrichment of immunostaining. NMJ regions with immunostaining > sarcomeric immunostaining signal + 2*SD were counted as positive (pos), all others as negative (neg). Data represent mean ± SEM (n = 4 muscles). C–D: Fiber diameter as a function of subsynaptic accumulation of myosin Va and PKA-RI. Data represent mean ± SEM (n = 4 muscles). (TIF) [file pone.0040860.s002.tif]

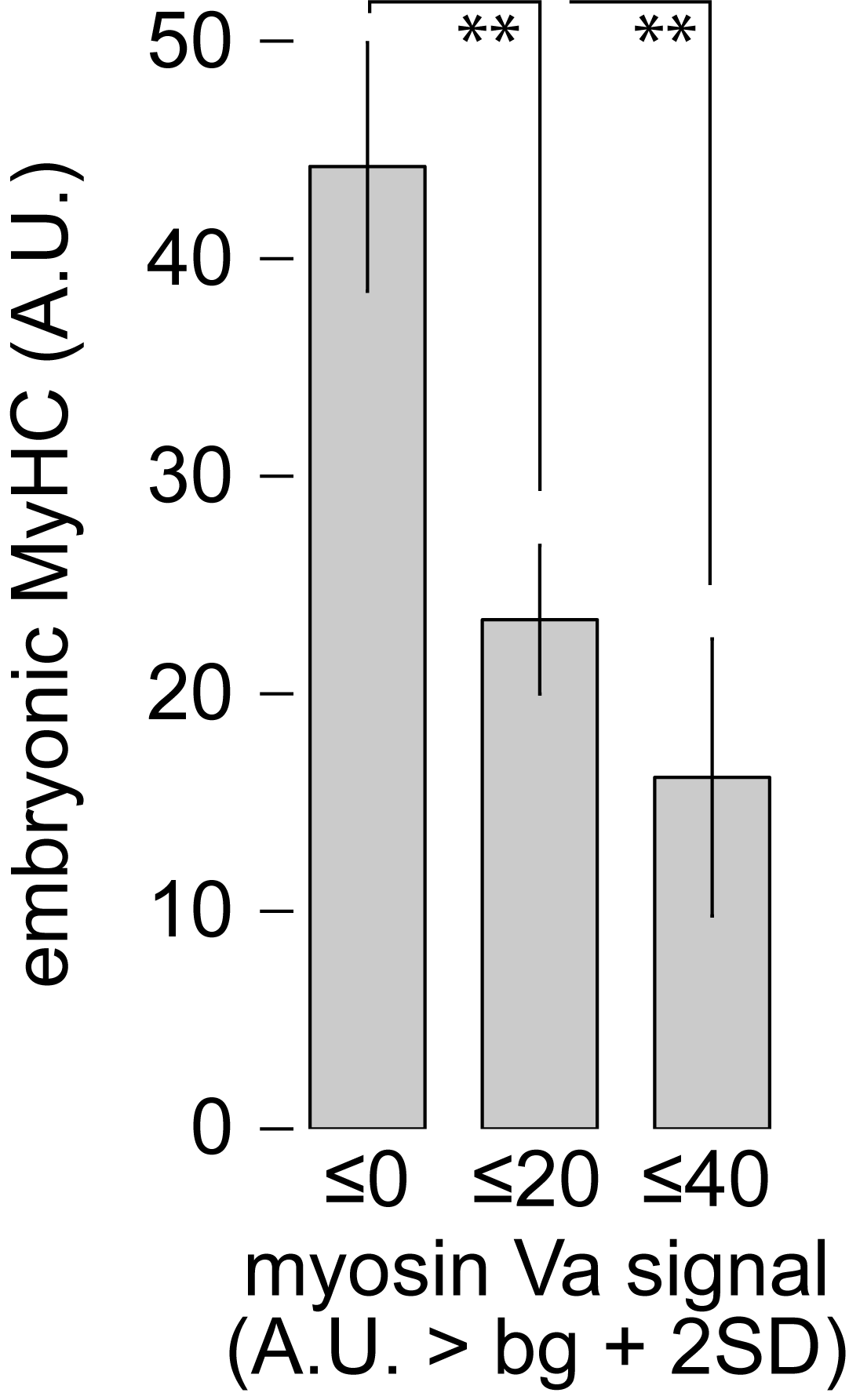

Supplement: Figure S3 — Embryonic myosin heavy chain expression inversely correlates with subsynaptic enrichment of myosin Va in regenerating EDL muscles. EDL muscles from adult wildtype mice were injected with Notexin. Ten days later, muscles were harvested, snap-frozen and then co-stained against embryonic myosin heavy chain and myosin Va. Synapses were labeled with BGT-AF555. Muscles were imaged with confocal microscopy and then quantitatively analyzed usinFg Image J. Depicted is the amount of embryonic myosin heavy chain staining intensity as a function of subsynaptic accumulation of myosin Va. Data represent mean ± SEM (n = 5). (TIF) [file pone.0040860.s003.tif]
